# Balancing everyday life—Patients’ experiences before, during and four months after kidney transplantation

**DOI:** 10.1002/nop2.225

**Published:** 2018-12-09

**Authors:** Charlotte Nielsen, Jane Clemensen, Claus Bistrup, Hanne Agerskov

**Affiliations:** ^1^ Department of Nephrology Odense University Hospital Odense Denmark; ^2^ Department of Clinical Research University of Southern Denmark Odense Denmark; ^3^ Centre for Innovative Medical Technology Odense University Hospital Odense Denmark; ^4^ HCA Research Hans Christian Andersen Children’s Hospital, Odense University Hospital Odense Denmark

**Keywords:** kidney, nurses, nursing, renal, transplantation

## Abstract

**Aim:**

To explore patients’ experiences before, during and four months after kidney transplantation as a coherent process.

**Design:**

A qualitative explorative study with a phenomenological‐hermeneutic approach.

**Methods:**

Participant observations and semi‐structured interviews were used complementary. In total, 18 kidney recipients were included. Data were analysed in accordance with Ricoeur's theory of interpretation, on three levels: naïve reading, structural analysis and critical interpretation and discussion.

**Results:**

Three themes emerged: waiting time and hope during everyday life, transformation during the kidney transplantation process and towards a new everyday life with positive prospects. Going through the kidney transplantation process was challenging for the patients. Everyday life was affected through the process, and the patients had to balance the associated challenges like hope, positive prospects and health‐related issues.

## 1 INTRODUCTION

This paper presents patients’ experiences during the period from approval for kidney transplantation to four months after transplantation. The knowledge gained provides a deeper understanding of transplantation as a coherent process from a patient perspective, which is significant to the improvement of treatment and nursing care during the process.

Kidney transplantation is, when possible, the treatment of choice for patients with end‐stage renal disease because of its survival benefit, higher quality of life, less medication and fewer restrictions in everyday life—compared with dialysis (Landreneau, Lee, & Landreneau, 2010; Oniscu, Brown, & Forsythe, 2005). Kidney transplantation is a significant milestone; however, everyday life may be affected, because transplantation involves continuous close contact with the healthcare system. Furthermore, being a kidney recipient implies daily routines, such as self‐monitoring, medical adherence, careful hygiene and sufficient fluid intake. A total of 543 individuals were enlisted for kidney transplantation and 257 were transplanted among these 165 with a kidney from a deceased donor in Denmark in 2017 (Scandiatransplant, 2018).

## 2 BACKGROUND

Patients hoped for a normal life after transplantation and had only positive expectations despite of not knowing what a kidney transplantation implied; however, the focus was specifically on recipients’ experiences of a challenging decision‐making and the relationship to the living donor in a literature review of patient experiences in the period before living donor kidney transplantation (Croft & Maddison, 2017). Studies of the time before transplantation are often aimed at patients waiting for a kidney from a deceased donor. Experiences during this period were characterized as an uncertain and confusing time with thoughts of the deceased donor. Furthermore, hope and expectations of a future free from dialysis treatment were present (Burns, Fernandez, & Stephens, 2017; Chong et al., 2016; Croft & Maddison, 2017; Spiers & Smith, 2016; Tong et al., 2015). Another perspective was how waiting time was used to prepare patients for the upcoming transplantation (Jones et al., 2016; Rodrigue, Mandelbrot, & Pavlakis, 2011; Sieverdes et al., 2015).

Postoperative discomfort such as pain and nausea was showed in a study of the first postoperative days among patients with a living donor; however, the main theme related to the relationship with the living donor (Bertelsen et al., 2015). Barriers towards learning caused by emotional reaction, side effects from medication and the physical changes were identified among kidney recipients in the period from the transplantation until six weeks after. An individual approach was desirable, to tailor information and develop individual everyday competences (Urstad et al., 2012; Wiederhold, Langer, & Landenberger, 2011).

Patients’ experiences after transplantation are well described, with a time range from six months until many years after. A need to improve self‐management and patient education has been identified to support stability in everyday life with the obligations of being a kidney recipient. One area of focus is the patient perspective on self‐management and education, to improve transplantation outcome and stability in everyday life. (Grijpma et al., 2016; Jaieson et al., 2016; Rosaasen et al., 2017; Schmid‐Mohler et al., 2014).

In summary, the systematic literature search identified patients’ perspective of kidney transplantation on specific stages of the transplantation process and selected topics, such as education or adherence have been covered. There is a lack of studies investigating patients’ experiences of going through transplantation as a coherent process. Therefore, to close the knowledge gap, patients’ experiences of what is significant during the process could provide valuable new knowledge for improving quality of treatment and nursing care to meet the patients’ specific needs.

### 2.1 Research question

How do the patients experience the process before, during and four months after kidney transplantation?

## 3 METHODS

The study was a qualitative explorative study of the patients’ experiences during the transplantation process. The approach applied was phenomenological‐hermeneutic inspired by Ricoeur's theory of narrative and interpretation. Ricoeur combines phenomenology with a critical hermeneutic philosophy, which makes it possible to reach a new understanding through critical interpretation (Pedersen, 1999; Ricœur, 1976). Semi‐structured interviews (Kvale & Brinkmann, 2014) and participant observations (Spradley, 1980) were performed. Interviews are reflected experiences told by the participants to the researcher (Kvale & Brinkmann, 2014). Participant observations are data collected by researchers participation in practice where observations reveal specific information that the participants might not express in words, including informal interviews which contribute with the patients’ perspectives to the observations (Spradley, 1980). The combination of the two methods provides a rich and valid data material to gain in‐depth knowledge of the transplantation process.

### 3.1 Setting

The study took place in one of three Danish kidney transplant centres, situated at a university hospital. The study included patients with living or deceased donors to achieve a rich data material. However, it was not the intention to compare the two groups. Approval for kidney transplantation included a consultation with a nephrologist after several clinical examinations and physical tests. Subsequently, patients were waiting for the approval of the donor or were referred to the waiting list. The patients were admitted acute or the day before the transplantation and hospitalized for approximately one week postoperatively. Afterwards, they came to the outpatient clinic for evaluation of their clinical condition and renal status. In the first four weeks, the patients attended the outpatient clinic twice a week. After the first month, the consultations became less frequent.

### 3.2 Participants

Participants represented patients from three different stages of the kidney transplant process: Those accepted for transplantation, patients undergoing transplantation and at four months after transplantation. The participants were screened using purposeful sampling according to gender, type of donor and age. Inclusion criteria were as follows: patients waiting for or undergoing a kidney transplantation, patients who speak Danish. Exclusion criteria were as follows: patients under the age of 18 or lack of mental acuity. In total, 18 patients were included: six women and 12 men, with a mean age of 53 years (Range: 33–73). The participants characteristics are presented in Table 1.

**Table 1:** Participants characteristics

Participant	Sex M: Male F: Female	Age Years	TX type L: Living D: Deceased	Participation O: Observation I: Interview	Stage B: Before D: During A: After
P1	M	57	L	O	B
P2	M	55	L	O	B
P3	M	33	L	O	B
P4	F	42	D	O	B
P5	M	49	D	I	B
P6	M	51	D	I	B
P7	F	67	D	I	B
				O	D
P8	M	42	L	O	D
P9	F	43	L	O	D
				I	A
				O	A
P10	M	68	D	O	D
P11	M	68	L	I	D
P12	M	61	D	I	D
P13	M	43	L	I	D
P14	F	39	L	I	D
P15	F	66	D	O	A
				I	A
P16	F	37	L	O	A
				I	A
P17	M	73	D	I	A
P18	M	57	D	O	A

**Figure 1:**
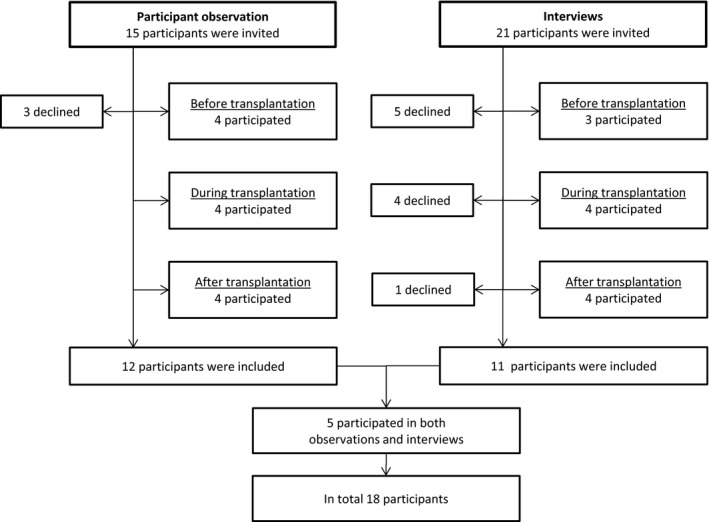
Inclusion of participants for participant observations and interviews

Participants were included in participant observation or interviews. Among 15 patients invited, 12 accepted participation in participant observations. Three pre‐transplant patients declined participation, because they found it difficult to talk about their situation. Among 21 patients invited, 11 participated in individual interviews. Due to lack of mental and physical resources, five patients waiting for a kidney and four patients from the early postoperative stage declined participation. Furthermore, one interview with a posttransplant patient was not completed due to patient withdraw. Five patients participated in both participant observations and interviews. The inclusion of participants is illustrated in Figure 1.

### 3.3 Data collection

Data were collected by the first author from April to November 2016. The first author is experienced in qualitative research and renal care, however, not involved in clinical work.

### 3.4 Before transplantation

Participant observation was performed by following four participants, during a three‐day clinical examination programme of evaluation for transplantation at the hospital. In addition, interviews were performed with three participants, allowing them to tell about their experiences of being on the waiting list. The interviews were planned to take place before their transplants; however, two participants received their transplant before the interview took place at the hospital.

### 3.5 During the time close to transplantation

Four participants were followed with participant observation on the ward before and after the transplant operation and during the first outpatient consultations. In addition, four participants were interviewed about their experiences. The interviews took place in the homes of the participants or at the hospital, approximately five weeks posttransplantation.

### 3.6 After transplantation

Finally, participant observation was conducted at the outpatient clinic with four participants approximately four months after the kidney transplantation. Subsequently, three of them were interviewed in their homes about the experiences they had following the transplantation. Because of patients’ restitution and time limitations, data were collected four months posttransplantation.

Participant observation was conducted with inspiration from Spradley's nine domains, as a support to the structure and to help keep an open mind during the observations (Spradley, 1980). Field notes were taken during observations and transcribed immediately afterwards. Field notes contained the researcher's description of the observations and short quotations from informal interviews. In total, 150 hr of participant observation were conducted. All interviews were performed using a semi‐structured interview guide with open‐ended questions that provided the opportunity to let the participants narrate about their experiences, such as: “Please, tell me about your experiences of the time close to your kidney transplantation.” Literature and data from the participant observations were used to develop the interview guide. The interviews lasted 18–83 min and were recorded and transcribed verbatim by the first author. The findings were discussed by the entire research team.

### 3.7 Data analysis

**Figure 2:**
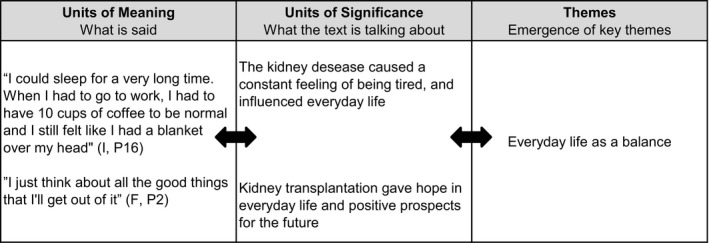
Illustration of structural analysis

The field notes and the transcribed interview data were analysed as one coherent text. Inspired by Ricoeur's thoughts about narrative and interpretation, the analysis was conducted on three levels: naïve reading, structural analysis and critical interpretation and discussion (Pedersen, 1999; Ricœur, 1976). The naïve reading is the first impression of the text, where the researcher reads the text with an open mind to “let the text speak” and the first impressions are obtained. In the structural analysis, there is a movement from the participants’ quotations, that is, “what is said” to a first interpretation of “what the text is talking about.” It is a dialectical process, in which themes will emerge and continuously be compared to the text and the impressions in the naïve reading to fulfil a deeper understanding and interpretation argued with the text itself (Pedersen, 1999; Ricœur, 1976), as illustrated in Figure 2.

It is the beginning of a movement from an individual to a general level, which will further unfold in the final critical interpretation and discussion of the emerged themes. A significant approach is the distance to the text, which makes it possible for the researcher to work thoroughly with the text to get in‐depth knowledge and achieve a new understanding of the investigated field (Dreyer & Pedersen, 2009). The approach inspired by Ricoeur does not include patient evaluation to validate the interpretation. According to Ricoeur, narration implies a process of reflection in the participants and leads to new perspectives into their lives, which make validation among the participants inappropriate (Ricœur, 1976).

### 3.8 Ethical considerations

The participants were informed orally and in writing about the study, in accordance with applicable ethical rules (Helsinki, 2018). Participants gave written informed consent. The study was approved by the Danish Data Protection Agency, journal number: 15/48886. Approval from The National Committee on Health Research Ethics was not required, in accordance with Danish law.

## 4 RESULTS

The naïve reading revealed that the patients’ everyday lives were affected in different ways through the transplantation process. It emerged that there were experiences of challenges, hope and gratitude during the process. Through the structural analysis, three themes emerged: waiting time and hope during everyday life, transformation during the kidney transplantation process and towards a new everyday life with positive prospects. Table 2 gives a presentation of themes and subthemes. The themes will be interpreted in the following section. (I) refers to interview, (F) refers to field note and (P) refers to the participant number.

**Table 2:** Themes and subthemes derived through the structural analysis

Theme	Subthemes
Before	
Waiting time and hope during everyday life	Everyday life as a balance
	Opposing reflections before transplantation
During	
Transformation during the kidney transplantation process	Dealing with being a patient at the hospital
	Balancing between recovery and ensuring that the kidney graft functions
After	
Towards a new everyday life with positive prospects	

### 4.1 Waiting time and hope during everyday life

Before the kidney transplantation, the patients focused on everyday life, but there were also reflections on the future transplantation.

#### 4.1.1 Everyday life as a balance

Living with end‐stage renal disease caused symptoms that affected everyday life, as one expressed:

“I could sleep for a very long time. When I had to go to work, I had to have 10 cups of coffee to be normal and I still felt like I had a blanket over my head” (I, P16).

The experience was a constant feeling of being tired. It also had a mental influence. Hence, the kidney disease caused difficulty concentrating and need for rest during the day. The limited resources influenced the way roles and tasks could be managed in everyday life. Often, the patients’ limited resources meant that they could not fulfil their own expectations. This could challenge family and working life, because resources had to be balanced. Nutrition, fluid and dialysis treatment could also cause restrictions to everyday life.

Restrictions in everyday life gave strength to the hope for kidney transplantation, as illustrated in this field note, with a quotation:

The patient relates about what he considers he will get out of the transplantation; first and foremost, freedom to travel but also in his everyday life […] “I just think about all the good things that I’ll get out of it” (F, P2).

This expresses hopeful thoughts about having a kidney transplant. There was a focus on positive prospects and this could be a way to maintain hope in everyday life. The thoughts of transplantation were positive and optimistic and possible disadvantages and complications were not addressed as an issue. Hence, everyday life implied balancing the kidney disease and the hope for transplantation.

#### 4.1.2 Opposing reflections before transplantation

The time before the transplantation was perceived variously, depending on the prospects of having to wait for a deceased or living donor. One patient with a living donor expressed:

“I had known for a long time that I was going to have a transplant [...] from May [one year ago] I started to train really hard to be ready” (I, P13).

This illustrates how the time before transplantation was used as preparation, in the knowledge that there was a living donor. The prospect of a limited timeframe before transplantation resulted in self‐preparation. This could be physical, that is, improving one's health; however, there was also mental preparation, alone or together with the donor, by sharing thoughts about the future transplantation.

In contrast, the situation seemed different when waiting for a deceased donor, as illustrated in this quotation:

“You can’t go round wondering about it every day, or else you can’t live your life” (I, P17).

The waiting time for a kidney from a deceased donor was of unknown duration and it could last for years. The focus was on everyday life. Thus, the transplantation was placed in the background. Nevertheless, there was still a belief that the transplant would succeed one day:

“Of course, it’s going to be my turn at some point [...] I’m delighted every time someone gets a transplant” (I, P5).

Knowing about other patients’ transplantations generated hope and there was a complete understanding of how the waiting list worked, that is, that it was based on finding the best match, rather than being operated on a first come first served principle. The time before the transplant revealed significant opposing experiences due to the type of donor and time perspective for transplantation. It involved either a focus on preparation or on maintaining everyday life. Nevertheless, this period was the only time when the patients’ experiences were connected to the donor type.

### 4.2 Transformation during the kidney transplantation process

The patients underwent a transformative process—from being in need of a kidney transplantation to managing everyday life as a kidney recipient.

#### 4.2.1 Dealing with being a patient at the hospital

The days after the transplant were challenged by postoperative symptoms, such as tiredness, pain and immobilization:

“You lie in bed and sleep in the morning and wake up and eat something and then you sleep again and try to force yourself out of bed, but it’s almost impossible to get up on your own, because it tightens up the wound” (I, P12).

Days merged together and there was only sufficient energy to fulfil one's most basic needs. Thus, physical challenges after transplantation dominated the early postoperative days.

It could be difficult to understand the expectations of patients at the hospital, as written in a field note with a quotation:

The doctor asks if the patient has any questions: “No, if you’re happy then I’m happy” (F, P9).

The postoperative circumstances and the fact that the daily routines and expectations were not made explicit created uncertainty about expectations of them which, meant that it was the healthcare professionals who had to take the initiative and assess the patient's situation. The non‐transparent agenda and structure on the ward made it challenging to take part in daily routines.

During the course of admission, involvement in treatment and nursing care seemed to increase, as illustrated in a field note:

The patient looks at his morning medicine and says: “I have to take a phosphate supplement” and picks up a copy of his blood test results and talks about his blood tests. The nurse looks at the observation notes and the patient says what his weight is, because he has weighed himself that morning (F, P8).

This shows how the daily routine on the ward was followed by observing the healthcare professionals’ actions and behaviour. This facilitated motivation and initiative, as it became possible to take part in treatment and care. In contrast to the early postoperative days, the patients’ energy and motivation increased and this allowed them to show more interest in education. New questions were raised, related to medicine, hygiene, nutrition, wound care etc. Thus, during the course of the admission, there was a transformation from a need for care and for expectations to be made explicit, to a need for support and patient education.

#### 4.2.2 Balancing between recovery and ensuring that the kidney graft functions

Going home was an opportunity to create a new everyday life after discharge and it also meant taking care of the kidney on one's own:

“They all [the health professionals] say to ring, but I ring them only as a last resort” (F, P7).

Once home was followed by feelings of balancing increased responsibility for the kidney and the ability to assess one's own needs for support without troubling the healthcare professionals. The involvement in treatment and care had increased over the course of the admission; however, the responsibility was experienced as overwhelming once back at home on one's own. Being discharged was perceived as a milestone in the process:

“You feel good to be at home and it helps me to be relaxed [...] to get my own structure and everyday life, I mean, you don’t feel so good at the hospital” (I, P16).

Going home was experienced as a significant step, because recovery was best achieved at home. However, side effects after the kidney transplantation affected everyday life:

The patient relates that she is very happy to be home, even though she is very tired and is in pain [...] she sleeps badly, because she has to get up to go to the toilet every two hours (F, P7).

Despite the fact that it was good to be at home, physical and psychological side effects challenged everyday life and the feeling of being vulnerable emerged. Practical and emotional support was needed to facilitate recovery. New ways had to be developed to structure everyday life, regarding medical adherence and self‐monitoring. New habits were developed, for example, how to cope with nutrition, hygiene and medication. However, everyday life also involved positive changes:

“I'm looking forward to running and riding and all those things that I love and of course there are also a lot of limitations, but it's really just about setting some conditions” (I, P14).

Everyday life was a balance between coping with health‐related issues regarding being a kidney recipient and a new life with future possibilities. There was a need for being at home for recovery and adapting to new habits to be able to balance the everyday challenges and the new possibilities.

Conversely, there were also a need for contact to the healthcare system as the kidney graft functioning could fluctuate especially in the immediate posttransplant period. It affected everyday life, with worries about the new kidney and outpatient visits or admissions. Often, the need for kidney biopsy was discussed:

“For five weeks I discussed it with them, I didn’t want the biopsy, I was afraid because of the risk of bleeding [...] I was so scared” (I, P16).

The discussions about the kidney graft functioning and need for a kidney biopsy were stressful and resulted in vulnerability because of the possible complications and consequences. The intervention was described as a threat hanging over one's head. It involved being constantly on standby and ready to go to the hospital at any time, which compromised a relaxed recovery at home.

Consultations at the outpatient clinic were the connection between recovery at home and ensuring the kidney graft functioning. Appointments were taken seriously; however, patients perceived that it was the nephrologist who set the agenda. Various perspectives were expressed:

“I think it’s been great to find out about the blood test results and that they got better, that’s been reassuring” (I, P11).

In contrast, another expressed:

“If I’m required [to go in for a check‐up], then I’ll do it, [...] but when it’s going as well as it is, then I think it’s meaningless” (I, P11).

Thus, the outpatients visits provided confidence and ensured the kidney graft functioning. However, time spent on visits could be perceived as unnecessary if there were no health‐related problems. There was a need to balance between monitor the function of the transplanted kidney in cooperation with the healthcare professional and at the same time a need to stay at home for recovery.

### 4.3 Towards a new everyday life with positive prospects

Four months after discharge, everyday life began to be reestablished, as one expressed:

“Now I have the structure, the times to take medicine, what I have to eat and I have to drink three litres, now I have structure over my things, so suddenly it has started to be a normal everyday life that, that’s the way my life is” (I, P16).

A structure involving new routines, related to being a person with a kidney transplant, began to be a part of everyday life. However, tiredness was an ongoing issue, as one said:

“I’m still trying to improve and I’m not finished with that, I still want it to be even better” (I, P9).

Everyday life improved after the transplantation; however, rehabilitation was not fully achieved. There continued to be an expectation of improved health. As the focus on everyday life increased, the need for frequent contact with the healthcare system decreased:

“Sometimes they make adjustments to the pills and the doctor could do that here at the local hospital, if it came to that” (I, P17).

The participants’ confidence and belief in their ability to take care of themselves increased, leading to outpatients consultations were of less significant to the participants.

Overall, looking back four months after the kidney transplantation process, it was experienced as positive and expressed as a metaphor:

“Two months in hospital [because of complications] is nothing, because just think, I got my life back” (I, P16).

Going through the process was experienced as overwhelming and challenging; however, the outcome outweighed the challenges and provided the opportunity to live an everyday life with positive prospects due to improved health and new possibilities.

## 5 DISCUSSION

The study showed how reestablishment of everyday life was significant and that a balance was achieved between hope, positive prospects and health‐related challenges throughout the transplantation process. The challenges which the patients were balancing can according to Meleis et al. be explained as a multi‐faceted and complex transition process (Meleis et al., 2000). Various types, patterns and properties of transition were represented during the transplantation process and in accordance with the theory the patients’ challenges can be viewed as a balance between a healthy transition and vulnerability (Meleis et al., 2000).

Before transplantation, the kidney disease restricted everyday life; however, the prospect of a kidney transplant gave the patients hope. The patients with a living donor could prepare for the transplantation. Wait‐listed patients focused on everyday life and maintaining belief that, the transplant would succeed at some point in the future. In accordance with Meleis et al., a transition is a movement over time, which can be experienced as an ongoing long‐term transition similar to the patients’ situation before transplantation (Meleis et al., 2000). Our findings are similar to others, where waiting for a kidney is described as becoming a normalized part of everyday life and how hope is generated when other patients in the dialysis community receive a transplant (Burns et al., 2017). Several studies exploring the period pre‐transplantation focus on education whose aim is to help the patients to be prepared for their new situation (Jones et al., 2016; Rosaasen et al., 2017). Our study showed that wait‐listed patients could not relate to ongoing education regarding the future transplant. They moved the possible future transplant into the background and focused on maintaining everyday life. Studies find that the challenge of being a wait‐listed patient includes confusion, worries and constant uncertainty (Burns et al., 2017; Rosaasen et al., 2017; Spiers & Smith, 2016). In our study, wait‐listed patients experienced the same challenges and were not in a mental condition to cope with education. Thus, it could be significant to support everyday life as wait‐listed and facilitate hope, for example, knowing of others who received a transplant. In contrast, our study showed that patients with a living donor spent the time before the transplant on mental and practical preparation. It is mentioned how transplant from a living donor gives an opportunity for patient education (Crawford et al., 2017). We found how these patients were motivated for education related to the transplant process. To our knowledge, studies did not identify this specific need, however. This might be because the period before a transplant with a living donor is not defined as waiting time.

Going through a kidney transplantation represented a challenging time for the patients and they seemed unprepared on admission. However, during the course of the admission, they became more involved and began to take responsibility for their treatment and personal care. Only a few studies seem to explore the first days of the kidney transplant with a focus on patient experiences in this early postoperative period. The time is illuminated as challenging, with postoperative discomfort and a “feeling of being torn” between the positive prospects and worries related to the kidney transplantation (Bertelsen et al., 2015; Urstad et al., 2012; Wiederhold et al., 2011). This echoes the findings in our study, where patients balanced health‐related challenges and hope for new possibilities after the transplantation. According to Meleis et al., in our study, transplantation was a transition and the challenges could be viewed as a balance between healthy transition and vulnerability. An inhibitor for healthy transition could be the patients’ lack of knowledge at the time of admission; however, their involvement increased over time which could be viewed as a facilitator (Meleis et al., 2000). Transplantation requires a new learning process, similar to an apprenticeship, because of the changes experienced (Wiederhold et al., 2011). This supports the importance of the patients’ increased involvement in treatment and care during admission to manage everyday life after discharge and to ensure the best conditions for long‐term graft survival. The patients were admitted for up to 21 days (Wiederhold et al., 2011), while patients in our current study were discharged after approximately a week. Thus, short hospitalization stresses the importance of involvement of the patients during admission as quickly as possible.

Patients described that they looked forward to going home. However, the period after discharge was challenging, because it involved balancing between adapting to daily routines and worries about the functioning of the kidney graft. Meleis’ theory of transitions explains how the patients were experiencing several transitions during the transplantation process and could move either in the direction of health or towards vulnerability (Meleis et al., 2000). In a study by Urstad et al., a gap between knowing and practice is identified. At home, the patients find it difficult to use the knowledge gained during admission; thus, the feeling of control is challenged (Urstad et al., 2012). Furthermore, kidney transplantation brings instability to most areas of the recipients’ lives (Schmid‐Mohler et al., 2014). This may explain the challenges the patients in our study experienced in the early period after discharge and how it took time to re‐establish everyday life and stability. Thus, being home for recovery seems significant in the early period after discharge.

Worries about rejection and loss of graft functioning have been identified in several studies (Crawford et al., 2017; Jamieson et al., 2016; Schmid‐Mohler et al., 2014; Urstad et al., 2012; Wiederhold et al., 2011). Furthermore, frequent follow‐ups and re‐hospitalizations were challenging, frustrating and tiring and experiencing of complications were stressful (Crawford et al., 2017; Schmid‐Mohler et al., 2014). Nevertheless, overcoming complications strengthens the kidney recipients’ capacity to master health situations (Jamieson et al., 2016). Given that unstable kidney graft functioning and complications occur most frequently close to the time of the transplant, this is well‐known to be a challenging time. Nonetheless, according to Jamieson et al., the experienced challenges will improve over time. Due to the design of our study, this was not a specific finding, as the patients were followed only up to four months posttransplantation. We found that the patients, at that time, were beginning to adapt to new routines in everyday life and the need for frequent contact with the health system was decreasing. This was also found by Jamieson et al. (2016). Furthermore, they found that, after kidney transplantation, the patients try to free themselves from the role of being a patient and that continuous contacts with the health system reinforce the identity as a patient and this can lead to non‐adherent behaviour (Jamieson et al., 2016). However, that does not seem to be an issue in our study. Four months after discharge, the patients began to re‐establish everyday life, still striving to improve their condition and were focusing on adherent behaviour. According to Meleis et al., the patients’ situation could be explained as going towards a healthy transition as stability became a part of everyday life. A process indicator could be the patients’ development of confidence with their situation (Meleis et al., 2000). Rosaasen et al. find that patients expect they should live a normal life after the kidney transplantation. However, the chronic disease is still a reality, just with a new set of things to deal with (Rosaasen et al., 2017). This could explain the patients’ experiences of adapting to a new everyday life at this point in the process.

### 5.1 Limitations

It can take up to a year or more before patients are completely familiar with their new everyday life situation (Crawford et al., 2017; Rosaasen et al., 2017; Schmid‐Mohler et al., 2014). This might have influenced our findings; however, our study showed that patients experienced that everyday life was beginning to be established four months after transplantation. Another limitation could be that, we did not follow the same participants throughout the transplant process. Instead, the participants were recruited from different stages of the process, contributing with their perspectives of the transplant process. Between four and eight participants represented each of the stages—before, during and after the kidney transplant. However, they could often contribute with experiences from more than one stage, depending on how far they were in the process. Thus, the study is based on experiences from 18 participants in total.

## 6 CONCLUSION

Exploring kidney transplantation as a coherent process provides a new understanding and insight into patients’ experiences. The transition perspective explained the coherent process as multi‐faceted and complex with various transitions, where patients are balancing hope, positive prospects and health‐related challenges. This can guide nursing care to recognize and support the patients’ individual needs in the kidney transplantation process.

## CONFLICT OF INTEREST

No conflicts of interest have been declared by the authors.
